# The prevalence of anemia and iron deficiency among pregnant Ghanaian women, a longitudinal study

**DOI:** 10.1371/journal.pone.0248754

**Published:** 2021-03-24

**Authors:** Ruth A. Pobee, Jacob Setorglo, Moses Klevor, Laura E. Murray-Kolb

**Affiliations:** 1 Department of Nutritional Sciences, The Pennsylvania State University, University Park, Pennsylvania, United States of America; 2 Department of Clinical Nutrition and Dietetics, University of Cape Coast, Cape Coast, Ghana; Addis Ababa University, ETHIOPIA

## Abstract

**Background:**

Gestational iron deficiency (ID) can be deleterious to mother and fetus. However, iron status is not routinely measured during pregnancy in Ghana. Therefore, the scope of ID in this population is unknown.

**Objective:**

To determine the prevalence of anemia and ID across pregnancy in the Central Region of Ghana.

**Methods:**

Women were recruited during their 1^st^ trimester of pregnancy (< 13 weeks; n = 116) and followed through to their 2^nd^ (n = 71) and 3^rd^ (n = 71) trimesters. Data on socio-demographic variables, weekly intake of iron-rich foods and vitamin C-rich fruits were collected. Blood samples were drawn and the concentrations of hemoglobin (Hb), ferritin (Ft), serum iron (sFe), total iron binding capacity (TIBC), were measured; transferrin saturation (TSAT) was calculated. Repeated measures ANOVA was used to determine change in anemia and iron variables over time with groups categorized by 1^st^ trimester iron status.

**Results:**

Participants were 27.1 ± 5.2 years, on average. Prevalence of anemia (Hb <11.0 g/dL) was 37%, 63%, 58%; ID (Ft <15 μg/L) was 16%, 20%, 38%; and iron deficiency anemia (IDA; based on low Ft and Hb) was 6%, 12%, 25% in 1^st^, 2^nd^ and 3^rd^ trimesters, respectively. Significant changes in Hb, Ft and TIBC occurred across time. Iron status at 1^st^ trimester had a significant effect on 2^nd^ but not 3^rd^ trimester iron status.

**Conclusions:**

ID is prevalent in pregnant Ghanaian women, especially during the 3^rd^ trimester. Anemia is a major public health problem during pregnancy in Ghana with a significant proportion due to factors other than ID.

## Introduction

Iron deficiency (ID) is known to be the single most prevalent nutrient deficiency in the world, affecting about 2 billion people worldwide [1]. Women-of-reproductive age (WRA) and children are the most vulnerable to ID. The United Nation’s Children Fund (UNICEF) estimated that globally, 50,000 young women die each year in pregnancy and childbirth due to iron deficiency anemia (IDA), and about 40 million pregnant women suffer from ID [2]. In high-income countries, data exist on the prevalence of ID due to the assessment of specific iron status biomarkers (ferritin, transferrin saturation, total iron binding capacity and transferrin receptor) in pregnancy [3]. However, in many low- and middle-income countries, including Ghana, such assessments are not part of routine care and, therefore, data on the prevalence of ID during pregnancy are not available. Instead, ID is estimated using anemia prevalence (hemoglobin (Hb) status) as a proxy, when in fact about half of anemia is attributed to ID [4]. Data show that a wide range of ID [1, 4–7] and ID anemia (IDA) [1, 4, 6] exists among pregnant women irrespective of the country of origin. The World Health Organization (WHO) estimates that about 38% of pregnant women are anemic worldwide, with 18% in high-income countries and 35–75% in low- and middle-income countries. The rate of deficiency seems to increase as pregnancy progresses. The most recent Ghana Demographic and Health Survey (GDHS) estimates anemia prevalence to be 45% among pregnant women [8].

The demand for iron increases during pregnancy. This high demand is necessary for expansion of plasma volume, increase in red cell mass, growth of the fetal placental unit, and to fulfill the fetal iron requirements [9]. If maternal iron stores are severely depleted, this can lead to anemia, which may impair the oxygen delivery to the placenta and fetus, thereby interfering with intrauterine growth and leading to preterm delivery and low birth weight [4, 6]. The deficiency has also been associated with both maternal and infant mortality [10].

Maternal ID during pregnancy has further consequences for fetal brain function and development [11]. Iron accumulation in brain cells takes place early during fetal development [6]. As such, gestational ID may lead to irreversible damage to brain cells, causing poor child cognition, poor motor development, behavioral abnormalities [1, 6] and maternal emotional disturbances [12]. The aim of this study was therefore to assess the prevalence of ID and anemia among pregnant Ghanaian women in their 1^st^, 2^nd^ and 3^rd^ trimesters. We hypothesized that the prevalence of ID and anemia would increase through the course of pregnancy.

## Methods

### Study design

A longitudinal study was carried out from October 2017 to September 2018. Women who were <13 weeks of gestation were recruited when they reported for antenatal care at health care facilities in the Central Region of Ghana. Multistage sampling was adopted in selecting the health care facilities. Initial data on antenatal attendance throughout the year at the health facilities were obtained from the regional Ministry of Health office. Health facilities that had previous antenatal attendances that were in-line with our research needs were purposefully selected. This yielded nine facilities. Simple random sampling was then used to select the health facilities used for the study. These facilities included the Moree Clinic, Cape Coast Teaching Hospital, the Cape Coast Metropolitan Hospital, Ewim Polyclinic, University of Cape Coast Hospital, Elmina Urban Health and Abura Dunkwa District Hospital. The nurses on duty informed prospective participants about the study and interested pregnant women were directed to the research team.

### Screening and recruitment

A brief written screening form was used to determine eligibility, which included attendance at any of the seven selected prenatal clinics in Central Region of Ghana; aged between 18–38 years old at enrolment; <13 weeks gestation at enrolment (determined by last menstrual period or ultrasound scan); expecting a singleton pregnancy with no known congenital anomalies; and no known history of diabetes mellitus or hypertension. Eligible and interested participants completed the consent form (which was read to each participant, since some of the women had no formal education) and were recruited. Upon written informed consent (either providing a signature or a thumb print), socio-demographic characteristics, anthropometric and blood pressure assessments, and venous blood sample draw occurred immediately for the first trimester visit, unless the participant requested to come back at a later date. Four trained field assistants carried out data collection. After the first visit was completed, each participant was provided with a date for her 2^nd^ trimester visit. Participants were then followed into their 2^nd^ (13–27 weeks) and 3^rd^ (28–36 weeks) trimesters. At each health facility, a nurse was recruited to coordinate activities between patients and the trained data collectors. At the end of the first two visits, each woman received a bar of soap plus transportation cost as incentive, and at the end of the third visit, each woman received a baby onesie plus transportation cost.

### Data collection procedure

Samsung Galaxy tablets were used to collect socio-demographic information including age, marital status, parity, education level, income and employment status. Dietary intake of vegetables, fruits and iron-rich foods, during the week prior to data collection, were also assessed at each visit.

#### Blood draws

Approximately 4 mL of blood were taken from the participant at each of the three trimesters during pregnancy by a trained phlebotomist at each health facility. Hb levels were determined on the spot via Hemocue (HB201; HemoCue America, Brea, CA, USA). The blood samples were stored on ice and delivered to the Cape Coast Teaching Hospital within 30 minutes of collection. Blood samples were centrifuged and serum aliquoted by a laboratory technician at the Cape Coast Teaching Hospital and subsequently stored in a -80^0^ C freezer prior to shipping to The Pennsylvania State University, USA, where iron status biomarkers (serum iron (sFe), total iron binding capacity (TIBC) and serum ferritin (Ft)) and inflammatory markers (alpha-1-acid glycoprotein (AGP) and c-reactive protein (CRP)) were determined in Dr. Murray-Kolb’s laboratory. Ft was determined via ELISA (Ramco Laboratories TX, USA), calibrated against WHO standards. sFe and TIBC were determined using colorimetric methods [13]. Transferrin saturation (TSAT) was calculated as (sFe/TIBC)×100. AGP and CRP were measured using radial immunodiffusion tests (Kent Laboratories Inc., Bellingham, WA, USA) and used to adjust Ft concentrations when inflammation was present. Ft values reported have therefore been adjusted for inflammation based on Thurnham criteria [14].

#### Follow-up visits for 2^nd^ and 3^rd^ trimesters

During the 2^nd^ (13–27 weeks) and 3^rd^ (28–36 weeks) trimester visits, all assessments, measurements and blood sample collection were repeated as described above, with the exception of socio-economic status (SES) assessment (which was only administered at the first visit).

### Ethical approval

Ethical approval was obtained from the ethical review board of the Ghana Health Service Ethical Review Committee, University of Cape Coast Institutional Review Board, Cape Coast teaching Hospital Ethical Review Committee and The Pennsylvania State University Institutional Review Board.

### Statistical methods

Statistical Analysis Software (SAS) version 9.4 (SAS Institute, Inc., Cary, NC, USA) was used for data analysis. Univariate analyses were run for all variables with the appropriate transformations applied to normalize all non-normal variables. The prevalence of ID was determined at each trimester. Proportions were presented for all sociodemographic (SES, marital status, age, years of schooling, income, parity) variables and iron supplement intake variables, and differences between the proportions of pregnant women who were iron deficient across the three trimesters were determined. One-way ANOVA and repeated measures ANOVA were used to determine significant differences between iron biomarkers over time and for change in iron variables over time based on 1^st^ trimester iron status, respectively. Simple regression models were used to determine if gestational age predicted iron status, adjusting for prenatal supplement intake.

## Results

### Sample population

**Fig 1:**
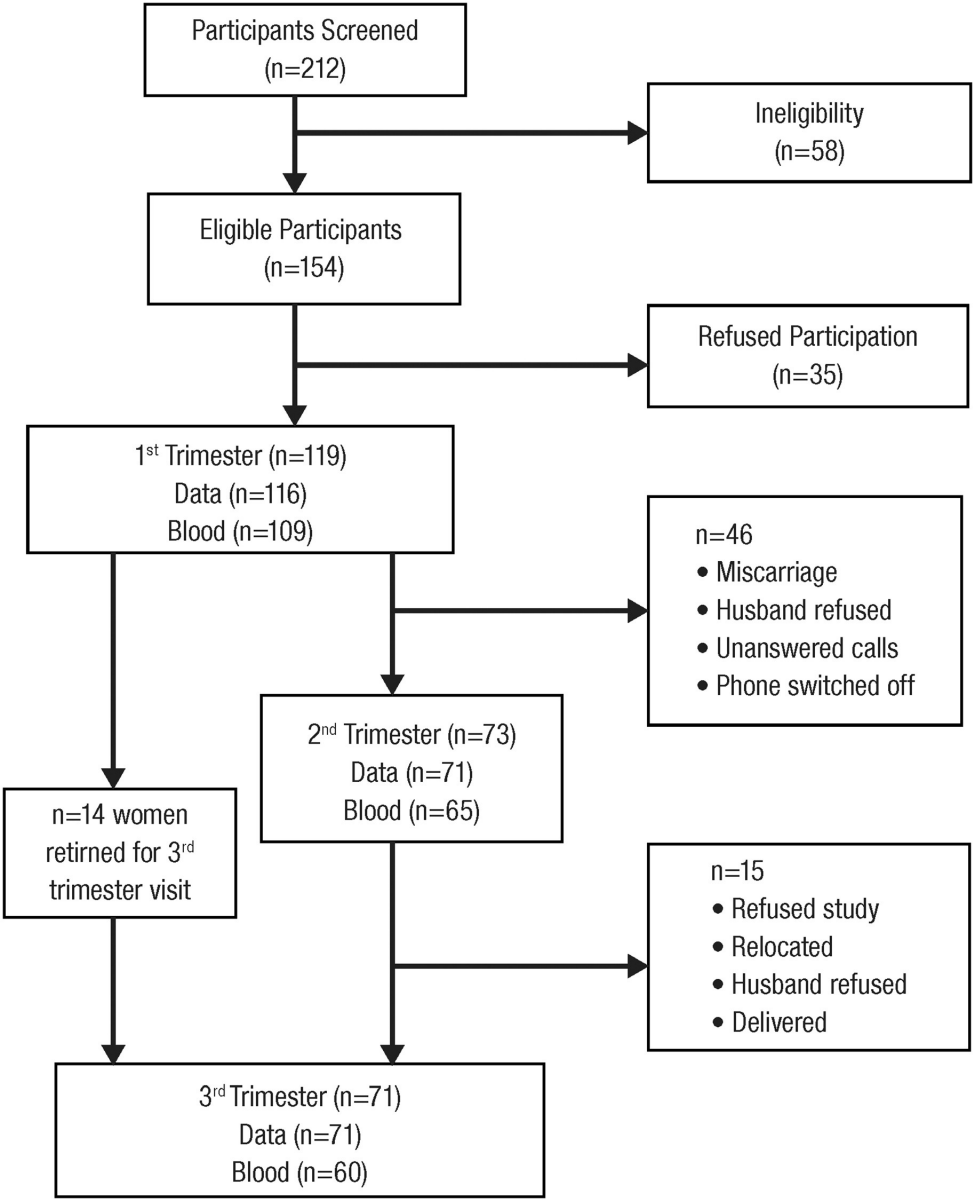
Participant flow chart by trimester.

Two hundred and twelve pregnant women were screened, out of which 154 were eligible (Fig 1). Thirty-five women refused participation so a total of 119 participants were recruited in their first trimester. Out of those recruited, 46 pregnant women dropped out of the study due to reasons such as miscarriage, spouse refusal, and unreachable (recipient out of coverage area and unanswered phone calls). Seventy-three pregnant women were followed into their 2^nd^ trimester. In the 3^rd^ trimester, 72 pregnant women were followed with 15 pregnant women dropping out between 2^nd^ and 3^rd^ trimesters due to reasons such as relocation, delivery and refusal to continue participation. Fourteen pregnant women from their 1^st^ trimester who were absent during the 2^nd^ trimester visit came back for the 3^rd^ trimester visit. The number of participants with complete socio-demographic and intake data were 116, 71 and 71, while 111, 68 and 65 participants provided a blood sample for 1^st^, 2^nd^ and 3^rd^ trimesters, respectively. After removal of outliers and missing blood concentrations (not enough blood to measure every biomarker), 109, 65 and 60 participants had values for all iron biomarkers for 1^st^, 2^nd^ and 3^rd^ trimesters, respectively (Fig 1).

### Baseline characteristics of pregnant women

The baseline socio-demographic characteristics of the pregnant women are shown in Table 1. The majority of the women (56.9%) were recruited from the Komenda Edina Eguafo Abirem (KEEA) District, specifically from Elmina Urban Health Center, with the least recruited from the Abura Asebe Kwamankese District (12.9%). The average age of participants was 27 years with 26% having BMI in the overweight range and 11% classified as obese (Table 1). About 68% of participants were married with 80% living with their husbands/partners or co-existing. About 17% of these women were heads of their households, meaning they were the sole breadwinners of their household. About 8% of the women had no formal education, while 45% had up to a middle school education, with only 10% having a university degree. In terms of income, 79% of these women had an income-generating activity, and the majority of these women (84%) earned between 500–1000 Ghana cedis per month (equivalent to US $125-$250). The majority (74%) of these women had national health insurance. Forty-seven percent of participants had children <5 years of age in their households, with 36% having two children <5 years in the household. The majority of participants (70%) had between 1–5 children, while 30% had no child prior to their current pregnancy. About 5% of the pregnant women had sickle cell traits.

**Table 1:** Socio-demographic characteristics of pregnant Ghanaian women in their first trimester (n = 116).

Sociodemographic variables	n	%
Age (yrs)	116 (18–38)*	27.1(5.2)#
BMI (kg/m^2^)	116 (16.2–40.2)*	24.6 (4.8)#
Underweight (<18.5)	6	5.2
Normal (18.5–24.9)	67	57.8
Overweight (25.0–29.9)	30	25.9
Obese (≥30.0)	13	11.2
Marital Status		
Married	79	68.1
Not Married	37	31.9
Lives with a partner/husband		
Yes	93	80.2
No	23	19.8
Head of household		
Yes	23	17.2
No	93	82.8
Educational Level		
No school	9	7.8
Primary	17	14.7
Middle	52	44.8
Secondary	26	22.4
University	12	10.3
Years of schooling		
0–6	12	11.2
7–9	56	52.4
>9	39	36.5
Income generating activity		
Yes	92	79.3
No	24	20.7
Income level (Ghana Cedis)		
GH 501–1000	97	83.6
GH 1001–2000	14	12.1
GH 2001–3000	3	2.6
GH 3001–5000	2	1.7
Valid health insurance		
Yes	75	74.3
No	26	25.7
Children 17 yrs and below in household		
0	40	34.5
1–4	66	56.9
>4	10	8.6
Children <5 yrs in household		
Yes	55	47.4
No	61	52.6
Number of children <5 yrs		
1	32	58.2
2	20	36.4
>2	3	5.5
Parity		
0	35	30.2
1–5	81	69.8
Sickle cell trait		
Yes	6	5.2
No	107	92.2
Unknown	3	2.6

*n (range),

^#^mean (SD)

### Prevalence of iron deficiency and inflammation

The prevalence of anemia (Hb <11.0 g/dL) was 37%, 63%, 58%; ID (Ft <15 μg/L) was 16%, 20%, 38%; ID (TIBC ≥ 400 μg/L) was 19%, 29% and 40%; ID (TSAT <16%) was 12%, 9% and 17%; iron deficiency anemia (IDA) based on Ft <15 μg/L and Hb <11.0 g/dL was 6%, 12% and 25% for 1^st^, 2^nd^ and 3^rd^ trimesters, respectively (Table 2). About 30%, 22% and 32% of pregnant women had inflammation based on CRP (≥ 5.0 mg/L) while 29%, 6% and 2% had inflammation based on AGP (≥ 1.0 mg/L) for 1^st^, 2^nd^ and 3^rd^ trimesters, respectively.

**Table 2:** Prevalence of iron deficiency in pregnant Ghanaian women and mean difference in iron biomarkers by trimester.

	1st Trimester (n = 109)*		2nd Trimester (n = 65)*		3rd Trimester (n = 60)*	
**Iron Biomarkers**	**n (%)**	**mean ± SE**	**n (%)**	**mean ± SE**	**n (%)**	**mean ± SE**
Hemoglobin, <11 g/dL	40 (36.7)	11.12 ± 0.13^a^	41 (63.1)	10.56 ± 0.16^b^	35 (58.3)	10.49 ± 0.17^b^
Serum Iron, <60 μg/dL	18 (17.0)	101.79 ± 3.97^a^	6 (9.2)	106.12 ± 5.07^a^	8 (13.3)	109.45 ± 5.28^a^
Adjusted Ferritin, <15 μg/L^†^	17 (15.6)	84.94 ± 5.55^a^	13 (20.0)	40.75 ± 7.19^b^	23 (38.3)	27.59 ± 7.49^b^
TIBC, ≥400 μg/dL	20 (18.9)	352.23 ± 8.06^a^	19 (29.2)	366.06 ± 10.29^a,b^	24 (40.0)	387.17 ± 10.71^b^
TSAT, <16%	13 (12.3)	29.65 ± 1.68^a^	6 (9.4)	29.94 ± 1.60^a^	10 (17.2)	29.14 ± 1.68^a^
**Iron and Anemia Categories**	**N**	**%**	**N**	**%**	**N**	**%**
IDA (Hb <11 g/dL & Ft <15 μg/L)	7	6.4	8	12.3	15	25.0
IDNA (Hb ≥11 g/dL Ft <15 μg/L)	10	9.2	5	7.7	8	13.3
ISA (Hb <11 g/dL & Ft ≥15 μg/L)	33	30.3	33	50.8	20	33.4
ISNA (Hb ≥11 g/dL & Ft ≥15 μg/L)	59	54.1	19	29.2	17	28.3
**Inflammatory Markers**						
CRP, Inflamed (≥5.0 mg/L)	33	30.3	14	21.5	19	31.7
AGP, Inflamed (≥1.0 g/L)	32	29.4	4	6.2	1	1.6
**Iron supplement intake**	**N (116)**	**%**	**N (71)**	**%**	**N (71)**	**%**
Yes	27	23.3	64	90.1	58	81.7
No	89	76.7	6	8.5	13	18.3
Don’t know	-	-	1	1.4	-	-
**Days iron supplements consumed/week**						
1 time	1	3.7	-	-	3	5.2
2–3 times	-	-	-	-	-	-
4+ times	26	96.3	64	100	55	94.8
**Dietary Intake/week**						
**Red meat**						
none	69	59.5	35	49.3	28	39.4
1 time	20	17.2	12	16.9	8	11.3
2–3 times	19	16.4	9	12.7	6	8.5
4+ times	8	6.9	15	21.1	29	40.9
**Fruit intake (oranges, pineapples, mangoes, pawpaws)**						
none	5	4.3	1	1.4	3	4.2
1 time	8	6.9	5	7	8	11.3
2–3 times	28	24.1	22	31	24	33.8
4+ times	74	63.8	42	59.2	36	50.7
Refused	1	0.9	1	1.4	-	-

TIBC: Total Iron binding capacity; TSAT: Transferrin saturation; IDA: iron deficiency anemia; Hb: Hemoglobin; Ft: Ferritin; IDNA: iron deficiency non-anemic; ISA: iron sufficient anemia; ISNA: iron sufficient non-anemic; CRP: C-reactive protein; AGP: Alpha-1- acid glycoprotein.

Different superscripts within a row indicate values which are significantly different based on ANOVA analyses

^†^Ferritin was adjusted using Thurnham correction (14)

*N for analyses of iron biomarkers after removal of outliers and those who had missing iron biomarker values.

### Change in iron biomarkers over time

Significant changes occurred in Hb, Ft and TIBC across time (Table 2). No significant change was found for sFe or TSAT over time. The pattern of change varied depending on the iron biomarker in question. Women showed significantly higher Hb concentrations in their 1^st^ trimester (11.1 ± 0.1 g/dL) compared with 2^nd^ (10.6 ± 0.2 g/dL) and 3^rd^ (10.5 ± 0.2 g/dL) trimesters; 2^nd^ and 3^rd^ trimester Hb concentrations did not differ. Like Hb, Ft values decreased over time. Pregnant women in their 1^st^ trimester showed significantly higher Ft concentrations (84.9 ± 5.6 μg/L) than pregnant women in their 2^nd^ (40.8 ± 7.2 μg/L) and 3^rd^ (27.6 ± 7.5 μg/L) trimesters, even after adjusting for inflammation. There was no significant difference in Ft concentrations between 2^nd^ and 3^rd^ trimesters. Consistent with iron status decreasing over time when measured by Ft, there was an increase in TIBC over time. Women in their 1^st^ trimester showed a significantly lower TIBC concentration (352.2 ± 8.1 μg/dL) than women in their 3^rd^ trimester (387.1 ± 10.7 μg/dL). Even though trends increased over time, there was no significant difference in TIBC concentration between 1^st^ and 2^nd^ trimesters nor between 2^nd^ and 3^rd^ trimesters. In a linear regression model with gestational age as a predictor of iron biomarker (Table 3), gestational age significantly predicted Ft and TIBC concentrations after adjusting for the number of prenatal supplements consumed. A unit increase in gestational age was associated with a decrease in Ft by 3.73 μg/L (p = <0.001) and an increase in TIBC by 1.96 μg/dL (p = 0.041). Gestational age was not a significant predictor of Hb, sFe or TSAT after adjusting for the number of prenatal supplements consumed.

**Table 3:** Gestational age as a predictor of iron status across trimesters in pregnant Ghanaian women*.

Dependent Variable	Predictor	Parameter Estimate	p-value	Adjusted R^2^
Hemoglobin, g/dL	Gestational age	-0.010	0.465	-0.010
Adjusted Ferritin, μg/L^†^	Gestational age	-3.727	<0.001	0.252
Serum Iron, μg/dL	Gestational age	0.037	0.930	-0.014
TIBC, μg/dL	Gestational age	1.955	0.041	0.017
TSAT, %	Gestational age	-0.147	0.274	-0.003

*Adjusted for number of prenatal supplements consumed.

TIBC: Total Iron binding capacity; TSAT: Transferrin saturation

^†^Ferritin was adjusted using Thurnham correction (14)

### Change in iron status over time depending on iron status at 1^st^ trimester

**Fig 2:**
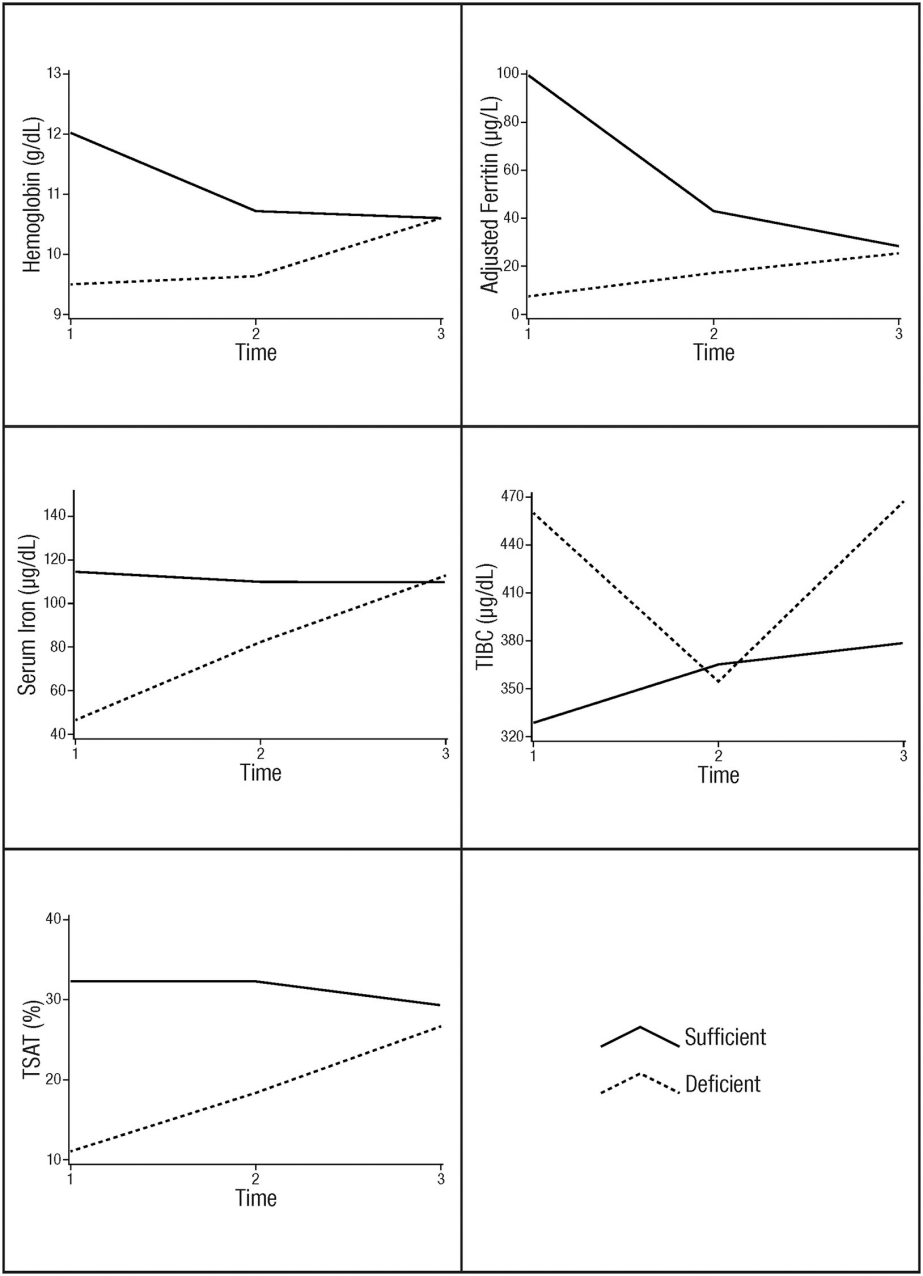
Change in iron status over time (1^st^, 2^nd^, 3^rd^ trimester) in Ghanaian women, categorized by 1^st^ trimester iron status.

There was significant interaction between all iron biomarkers (Hb, Ft, sFe, TIBC, TSAT) and time categorized by 1^st^ trimester iron status (S1 Table, Fig 2). Pregnant women who were non-anemic at 1^st^ trimester experienced a significant decrease in Hb concentrations by the 2^nd^ trimester (β = -1.22, 95% CI: [-1.63, -0.81]), with a further decrease by the 3^rd^ trimester (β = -1.58, 95% CI: [-1.99, -1.18]). However, women who started pregnancy anemic experienced a gradual but non-significant increase in Hb concentration by the 2^nd^ trimester and a significant increase by the 3^rd^ trimester (β = 1.18, 95% CI: [0.41, 1.95]) compared with the 1st trimester Hb concentrations. At the 3^rd^ trimester, Hb concentrations did not differ between women who were anemic and those who were non-anemic at 1^st^ trimester (Fig 2). For Ft levels, pregnant women who were sufficient in Ft at 1^st^ trimester had a significantly decreased Ft concentration by the 2^nd^ trimester (β = -57.02, 95% CI: [-78.98, -35.06]), with a further decrease by the 3^rd^ trimester (β = -67.40, 95% CI: [-86.77, -48.03]). On the other hand, pregnant women who had low Ft at 1^st^ trimester had an increase in Ft concentration by the 2^nd^ trimester, though not significant, and a significant increase by the 3^rd^ trimester (β = 18.04, 95% CI: [4.99, 31.09]); at the 3^rd^ trimester, ferritin levels were comparable between pregnant women who were Ft sufficient and those who were deficient in trimester one (Fig 2). For sFe, pregnant women who were deficient in the 1^st^ trimester increased sFe concentrations significantly across trimesters while those who were sufficient in sFe at 1^st^ trimester did not experience a change in their levels across trimesters. At the 3^rd^ trimester, women who were sFe deficient and those who were sufficient in the 1^st^ trimester had similar sFe levels. A similar trend was observed for TSAT. For TIBC, pregnant women with high levels in the 1^st^ trimester decreased their TIBC levels by the 2^nd^ trimester (β = -97.87, 95% CI: [-181.28, -14.45]), while sufficient women increased significantly in TIBC by the 2^nd^ (β = 37.92, 95% CI: [0.86, 74.98]) and 3rd trimesters (β = 46.53, 95% CI: [8.68, 84.40]).

## Discussion

In Ghana, not all biomarkers of iron status are routinely assessed during pregnancy and therefore the prevalence of iron deficiency in this population is unknown. We found that the prevalence of ID increased as pregnancy progressed with a high prevalence in the 3^rd^ trimester, as hypothesized. We found a significant time effect for Ft and TIBC concentrations, reflecting a pattern of ID which is commonly observed during pregnancy. As iron stores deplete, the amount of transferrin which is available to bind iron increases. Ft levels significantly decreased with gestational age while TIBC significantly increased with gestational age, as expected, during pregnancy.

A limited number of studies have measured iron status during pregnancy in Ghana with one reporting a prevalence rate of iron deficiency ranging from 5–46%, depending on the definition applied [15], while another reported a rate of 11% for women ≤24 weeks and 20% for women ≥36 weeks pregnant, with an overall prevalence of 16% during pregnancy [16]. We observed prevalence rates of 16%, 20% and 38% in the 1^st^, 2^nd^ and 3^rd^ trimesters, respectively. The major difference between these previously published studies and ours is that the previous studies were cross sectional while our study was longitudinal. Additionally, Mockenhaupt et al. [18] did not report prevalence rates by trimester and Engmann et al. [19] dichotomized gestational age into either ≤24 weeks or ≥36 weeks whereas we reported prevalence rates for 1^st^ (<13 weeks), 2^nd^ (13–27 weeks) and 3^rd^ (28–36 weeks) trimesters. Furthermore, Engmann et al. [19] used a population of women from the city and Mockenhaupt et al. [18] used a rural population; our population was predominately semi-urban. These differences may contribute to the various prevalence rates observed in our study compared to previous studies. A third study from Ghana which occurred in a comparable setting to the Central Region found the prevalence of ID to be 14%, 23% and 26% in the 1^st^, 2^nd^ and 3^rd^ trimesters, respectively [17]. Several measures of iron status were used, as in our study; however, the authors did not indicate the definition used to determine ID. Not only were cut-offs not reported, but, whether ID was based on one or multiple iron biomarkers was not indicated. Their rates in the 1^st^ and 2^nd^ trimesters were comparable to our 1^st^ and 2^nd^ trimester rates (which were based on Ft<15 μg/L) but our 3^rd^ trimester ID prevalence rate was higher (38%) than their 3^rd^ trimester prevalence of 26% [17]. As with the other two previous studies in Ghana, this study was cross sectional. Previous studies conducted in neighboring countries have reported higher ID rates during pregnancy. A study in Nigeria reported an ID prevalence rate of 48% [18], while a study in Malawi reported a prevalence rate of 32% [19] throughout pregnancy. Both studies were cross sectional, with the Nigerian study recruiting women at term while the Malawi study recruited only anemic pregnant women, thus excluding women who may have been iron deficient but not anemic.

When we compared the prevalence rates of ID found in our study to those in a high-income country such as the USA, we found a comparable pattern. Miller used the US NHANES 1999–2006 data and found an ID prevalence rate of 25.4% during pregnancy [20], while Mei *et al*. found ID prevalence of 7.3%, 23.7% and 29.2% for 1^st^, 2^nd^ and 3^rd^ trimesters, respectively [21] using a Ft cut-off of <12 μg/L. If we use the same cutoff of 12 μg/L for our population, we see a higher prevalence in the 1^st^ trimester (12%), a slightly lower prevalence rate in the 2^nd^ trimester (18%), and a comparable prevalence rate in the 3^rd^ trimester (30%). This suggests that ID prevalence among pregnant women in Ghana is comparable to rates observed in the US but lower when compared with rates from neighboring low- and middle-income countries. One likely explanation is the use of iron supplements, especially during the 2^nd^ and 3^rd^ trimesters. This is the standard of care in Ghana where pregnant women are given 30–60 mg of iron daily from the 2^nd^ trimester until delivery. If a woman is found to be anemic before this time point, iron supplements are prescribed upon the diagnosis of anemia. In our population, 23% took iron supplements in the 1^st^ trimester, while 90% and 82% took iron supplements in the 2^nd^ and 3^rd^ trimesters, respectively. Another possible explanation for prevalence rates that are lower than neighboring countries could be the high consumption of meat and fish in this population [22]. Our population is located along the coast with fishing as the main source of employment and fish as the main source of protein in these communities [23]. In our study, we found that the frequency of consuming red meat increased throughout pregnancy and that most of the women regularly consumed fruits with a high vitamin C content. Consumption of meat and fish along with foods containing a high vitamin C content likely reflects a diet with higher iron bioavailability compared to the diets of neighboring countries.

Despite the fact that these women were given iron supplements as routine standard of care, Ft levels decreased across pregnancy. Other iron biomarkers such as TSAT and serum iron did not change significantly over time, perhaps as a result of the iron supplements given as standard of care. The rate of anemia was high among the women studied. A rate of 37% in the 1^st^ trimester is regarded as a public health problem. Higher rates were observed in the 2^nd^ (63%) and 3^rd^ (58%) trimesters, both classified as severe public health problems.

The prevalence of IDA was 6%, 12% and 25% for 1^st^, 2^nd^ and 3^rd^ trimesters, respectively. Using the common assumption that half of anemia is due to iron deficiency [4], we would have expected rates of approximately 19%, 32%, and 29% for the 1^st^, 2^nd^, and 3^rd^ trimesters, respectively. Our lower than expected rates are an indication that much of the anemia in this population is due to factors other than iron deficiency. Although these rates are lower than those reported in several other countries [24], they are comparable to rates reported in other studies conducted in Ghana [19, 20]. These findings challenge the WHO estimation that half of all anemic cases can be attributed to ID and remind us that careful examination regarding the etiology of the anemia is warranted, before ID is assumed and supplements are dispensed. This is especially important in low- and middle-income countries where the etiology of anemia is complex and the risk for poor outcomes of iron supplementation as a result of underlying infections could be significant. In this population, we cannot rule out other causes of anemia such as hemoglobinopathies, nutritional deficiencies (such as folate, vitamin B12 and vitamin A), infectious diseases (e.g. tuberculosis and HIV/AIDS), parasitic infestations (e.g. hook worm) and malaria [25]. As such, before one begins to treat anemia, it is important to consider the various causes and to determine an appropriate diagnosis before supplementation is started.

When we examined changes in iron biomarkers over pregnancy, based on iron status in the first trimester, we found a significant effect of iron status at first trimester on 2^nd^ trimester iron concentrations but not on 3^rd^ trimester concentrations. Generally, pregnant women who were iron sufficient at 1^st^ trimester had a drop in iron concentrations but still maintained a higher iron status in the 2^nd^ trimester than those who were iron deficient in the 1^st^ trimester. However, by their 3^rd^ trimester, both those who started pregnancy deficient and those who started pregnancy sufficient in iron had comparable iron status. This likely reflects the physiology of pregnancy in the 3^rd^ trimester where maternal iron is quickly mobilized in order to meet the fetal transfer demands, and the provision of iron supplements during the 1^st^ trimester to women diagnosed with anemia but not given to all others until the 2^nd^ trimester of pregnancy.

The strengths of our study include the longitudinal design with the assessment of iron status at three time points, using multiple iron biomarkers during pregnancy and the fact that the population studied included individuals whose iron status is not routinely assessed during pregnancy, therefore adding needed knowledge to the literature. The main study limitation was the high dropout rate (37.8%) observed between 1^st^ and 2^nd^ trimesters.

## Conclusion

In Ghana, ID is prevalent during pregnancy, with the highest rates seen in the 3^rd^ trimester. Additionally, anemia remains a major public health problem during pregnancy in Ghana, and a significant proportion of anemia in this population is attributable to causes other than ID. Measures must therefore be put in place for thorough examination of anemia in pregnant women which should include assessment of iron biomarkers and not just Hb. This will help determine the cause of anemia before supplementation is started, which is especially important in countries like Ghana, where there are many potential causes of anemia.

## Supporting information

S1 Data (CSV)

S2 Data (CSV)

S1 Table Change in iron status over time categorized by 1st trimester iron status in Ghanaian women. (DOCX)

## Abbreviations

AGP: Alpha-1-acid glycoprotein
ANOVA: Analysis of Variance
CRP: C-reactive protein
Ft: Ferritin
Hb: Hemoglobin
ID: Iron deficiency
IDA: Iron deficiency anemia
SES: Socio-economic status
sFe: Serum iron
TIBC: Total iron binding capacity
TSAT: Transferrin saturation
UNICEF: United Nations Children’s Fund
WHO: World Health Organization
WRA: Women-of-reproductive age
