# Supplementary material for: The prevalence of anemia and iron deficiency among pregnant Ghanaian women, a longitudinal study
Source: PLoS One. 2021 Mar 24;16(3):e0248754. doi: 10.1371/journal.pone.0248754 (PMC7990185; doi:10.1371/journal.pone.0248754)
Supplement: S1 Table — (DOCX) [file pone.0248754.s003.docx]

**S1 Table. Change in iron status over time categorized by 1st trimester iron status in Ghanaian women**

TIBC: Total Iron binding capacity; TSAT: Transferrin saturation; Mean diff: Mean difference

Bold indicates significant difference compared to the reference

^†^Ferritin was adjusted using Thurnham correction (17)
